# Should we separately measure the pain parameter of the Constant-Murley score in patients with chronic shoulder pain?

**DOI:** 10.1186/s12891-023-06441-7

**Published:** 2023-05-19

**Authors:** Gil Augusto Pires Rodrigues, Philippe Vuistiner, Cyrille Burrus, Michel Konzelmann, Bertrand Léger, François Luthi

**Affiliations:** 1 Department for Musculoskeletal Rehabilitation, Department of Medical Research, Clinique Romande de Réadaptation SUVA, Avenue du Grand-Champsec 90, 1950, sion, Switzerland; 2 Musculoskeletal Department, Orthopedics and Traumatology Department, Lausanne University Hospital, Lausanne, Switzerland; 3 Department of Medical Research, Clinique Romande de Réadaptation SUVA, Avenue du Grand-Champsec 90, 1950, Sion, Switzerland; 4 Department of Medical Research, Assessment and Consultation Center, Clinique Romande de Réadaptation SUVA, Avenue du Grand-Champsec 90, 1950 Sion, Switzerland; 5 Department of Physical Medicine and Rehabilitation, Lausanne University Hospital, Rue du Bugnon 46, 1011, Lausanne, Switzerland

**Keywords:** Shoulder assessment, Psychological factors, Biopsychosocial approach, Chronic pain, Shoulder function

## Abstract

**Background:**

The Constant-Murley Score (CMS) is a relatively unique shoulder assessment tool because it combines patient-reported outcomes (pain and activity), performance measurement and clinician-reported outcomes (strength and mobility). With these characteristics, the effect of patient-related psychological factors on the CMS remains debated. We aimed to investigate which parameters of the CMS are influenced by psychological factors by assessing the CMS before and after rehabilitation for chronic shoulder pain.

**Methods:**

This retrospective study screened all patients (18–65 years old) who were admitted for interdisciplinary rehabilitation for chronic shoulder pain (≥ 3 months) between May 2012 and December 2017. Patients with unilateral shoulder injuries were eligible. Exclusion criteria were shoulder instability, concomitant neurological injuries, complex regional pain syndrome (including Steinbrocker syndrome), heavy psychiatric issues, and missing data. The Hospital Anxiety and Depression Scale, Pain Catastrophizing scale, and Tampa Scale of Kinesiophobia were administered before and after treatment. Regression models were used to estimate associations between psychological factors and the CMS.

**Results:**

We included 433 patients (88% male, mean age 47±11 years) with a median duration of symptoms of 392.2 days (interquartile range: 266.5-583.5). Rotator cuff issue was present in 71% of patients. During interdisciplinary rehabilitation, patients were followed for a mean of 33.6±7.5 days. The mean CMS at entry was 42.8 ±15.5. The mean gain in CMS after treatment was 10.6 ±10.9. Before treatment, psychological factors were significantly associated with only the pain CMS parameter: -0.37 (95% CI: -0.46 to -0.28), p <0.001. After treatment, psychological factors were associated with the evolution of the four CMS parameters: -0.12 (-0.23 to -0.01) to -0.26 (95% CI: -0.36 to -0.16), p<0.05.

**Conclusions:**

This study raises the question of a distinct assessment of pain when assessing shoulder function with CMS in patients with chronic shoulder pain. The separation of the “pain parameter” from the overall CMS score seems illusory with this tool that is used worldwide. However, clinicians should be aware that psychological factors can negatively influence the evolution of all CMS parameters during follow-up, which argues for a biopsychosocial approach to patients with chronic shoulder pain.

**Supplementary Information:**

The online version contains supplementary material available at 10.1186/s12891-023-06441-7.

## Introduction

Chronic musculoskeletal pain is a health problem with a worldwide prevalence ranging from 11.4 to 24% [1]. The recommended therapeutic approach combines physiotherapy, exercises, physical activity associated with therapeutic education, and cognitive-behavioral therapies in case of psychological distress, as part of biopsychosocial care [2, 3]. In addition, multiple treatments using physical or pharmacological approaches have been proposed [4–7]. Among the multiple sources of chronic pain [8], shoulder pain is one of the most frequent reasons for referral to health professionals. Clinicians use multiple tools to assess a patient with shoulder pain [9].

The Constant-Murley Score [10, 11] is one of the most commonly used assessment tools for various shoulder pathologies. Although it is not a gold standard, it has good psychometric properties and has excellent ability to detect clinically significant changes [10, 12]. It assesses four parameters: pain, activities of daily living, strength, and mobility. It is considered fairly user-friendly, although the measurement of strength requires some extra effort [13, 14]. The calculation of the score [15] is simple and easy. A higher score corresponds to better shoulder function.

The CMS is a relatively unique tool because it combines patient-reported outcomes (pain and activities of daily living: 35 points), performance measurement, and clinician-reported outcomes (strength and mobility: 65 points). Some consider this feature an advantage over patient-reported outcome measures (PROMs), which reflect only the subjective patient’s point of view, which are also influenced by psychological factors [16, 17]. Others consider that it represents a weakness and its interpretation is more complex [18]. The use of different tools (PROMs and observational measures) is considered a better option, although more cumbersome to implement. Moreover, the putative advantage of a single tool combining patient- and clinician-rated outcomes is no longer an argument if the different components of the CMS are also affected by psychological factors related to the patient (as are the questionnaires).

For many years, psychological factors have been considered associated with the patient’s perception of shoulder pain and disability in upper extremity injuries as well as the patient’s perception of the results of both surgery and physiotherapy [19–21]. The main factors with negative association are emotional (distress, anxiety, depression, preoperative concerns) and cognitive (fear-avoidance beliefs, pain catastrophizing, and low pain self-efficacy). Important outcomes such as pain, physical functioning, disability and quality of life are influenced by psychological factors.

For the CMS, the results remain conflicting. When determining to what degree shoulder outcome instruments reflect the patient’s psychological distress, Roh et al. found no such association with the CMS [22], whereas others found that the CMS and PROMs were influenced similarly by emotional (depression) and cognitive factors (pain catastrophizing and kinesiophobia) in patients with chronic shoulder pain [23]. This controversy needs to be clarified, as do the components of the CMS that could be affected: only patient-rated parameters (subjective: pain and activity of daily living) or also clinician-rated parameters (observational: mobility and strength). Should we determine whether such a correlation exists during patient follow-up?

Following these questions, the aim of our study was to investigate which parameters of the CMS are affected by psychological factors by determining the CMS before and after rehabilitation, here chronic shoulder pain. Depending on the results, a reflection on the interpretation and use of the CMS could be initiated. For instance, a more detailed knowledge of the psychological factors affecting each of the components of the CMS could promote a better interpretation of the score. The clinician could then be made aware of the detection of these emotional and cognitive factors, namely in case of discordance of the CMS with other evaluation parameters (clinical examination, imaging).

## Patients and methods

### Study design and setting

We conducted a single-center retrospective study in a Swiss rehabilitation teaching center. The protocol was approved by the local medical ethics committee (CCVEM 034/12). The study was performed in accordance with the ethical standards of the Declaration of Helsinki. Patients included underwent rehabilitation between May 1, 2012 and December 31, 2017.

### Participants/rehabilitation program

Patients with persistent shoulder pain and functional impairments from the French-speaking part of Switzerland were referred by surgeons, general practitioners, or insurance medical advisors [24]. All patients underwent an interdisciplinary rehabilitation. In Switzerland, work-related and non-work-related illnesses and accidents are covered by the same insurance, so there is no distinction in patient care and therefore no workers’ compensation programs.

All patients with persistent musculoskeletal complaints (≥ 3 months) after unilateral shoulder traumatic and nontraumatic injuries were eligible and were identified from electronic patient files. The exclusion criteria were concomitant neurological injuries, complex regional pain syndrome, heavy psychiatric issues (severe depression, personality, and somatoform disorders) assessed by a psychiatrist and missing data. We also excluded patients with persistent shoulder instability, the CMS being not appropriate for patients with shoulder instability[25]. If the patient had completed several rehabilitation stays, we considered only the first stay for our analysis.

The inpatient therapeutic program, involving a multidisciplinary biopsychosocial approach, lasted 4 to 5 weeks (at least 3 h of daily therapy, excluding weekends). This program consists of physical components (physiotherapy and occupational therapy) with individual and group sessions including graded exercise (strength and endurance training, stretching, balance, and adapted physical activities such as ball games, badminton etc.) and psychological components. The program included a mean of four psychological Cognitive-Behavioral Therapy (CBT) sessions as well as social advice and vocational training. After determining the baseline physical capacity of the patient, therapists determined the therapy and then adjusted after a weekly multidisciplinary meeting. Patients had the opportunity to alternate activity and rest according to their activity planning [26].

### Description of experiment

#### Measurement of the CMS

The primary outcome was the four parameters of the CMS before the therapeutic program began. A health professional scored the first two parameters (pain and activities of daily living) by interviewing the patient (subjective assessment) and the last two parameters (measurement of mobility and strength) by observational measurement. The secondary outcome was the improvement in score for the four parameters between the beginning and end of the therapeutic program.

#### Potential contributing factors

According to our previous study [23] and the Fear-Avoidance Model of pain [27], patients also completed three questionnaires to assess potential contributing factors:

The Tampa Scale for Kinesiophobia (TSK) [28, 29]. “Fear” related to pain often leads to avoidance of activities, considered to cause pain, called “kinesiophobia”. A series of clinical studies [27, 28] showed that kinesiophobia was a better predictor of disability than physical ability tests and pain severity tests. The Tampa scale was designed and validated to estimate the level of kinesiophobia. It contains 17 questions rated from 1 (strongly disagree) to 4 (strongly agree). A score of 17 is the lowest possible score and indicates no kinesiophobia, whereas a score of 68 is the highest possible score and indicates extreme fear of pain with movement.

The “Pain Catastrophizing Scale” (PCS) [30–32] screens for “catastrophist” patients who tend to focus on painful sensations, exaggerate the threatening aspect of pain and perceive themselves as being unable to control painful symptoms. This self-questionnaire consists of 13 items rated from 0 (not at all) to 4 (permanently) depending on the intensity felt by the patient. The final score can vary from 0 to 52, which represents the most catastrophist patients.

The Hospital Anxiety and Depression Scale (HADS) [33, 34] seeks to detect an anxious-depressive symptomatology and assess its severity. It has 14 items rated from 0 to 3. Seven questions relate to anxiety (HADS-A) and seven to the depressive dimension (HADS-D), which results in two scores (maximum of each score = 21). Higher scores indicate greater anxious or depressive symptomatology [35]. In our study, we used only the depression score because anxiety was not related, as observed in a previous study [23].

### Data collection

The CMS was determined by highly experienced physiotherapists who were familiar with the score via regular training and clear instructions [36]. To not influence the physiotherapists, the CMS was obtained before patients completed the questionnaires. To minimize measurement bias, data were collected before patients starting the therapeutic program, and all records (CMS and questionnaires) were collected electronically, without transcription from paper to data files. All questionnaires were completed by the patients at entry and at discharge.

### Statistical methods

Summary statistics are expressed as mean and SD for continuous variables, and number and percentage for categorical variables. To estimate the association between psychological factors at entry and the CMS parameters at entry and its evolution, we used linear regression models. All predictor variables (PCS, HADS-D, and TSK) were examined individually and adjusted for confounding variables. The following confounding variables were used: age, sex, pain severity, diagnosis (rotator cuff vs. others), surgery (yes/no), and work-related injury. Pain was assessed by the mean of the Brief Pain Inventory (BPI)[37, 38], a PROM that assesses pain severity and pain interference. It uses 11-point numeric rating scales (0 to 10) to assess pain severity (combining 4 subscales) and pain-related interference in seven dimensions. We used the pain severity score of the BPI for adjustment with the activity, mobility, and strength CMS parameters. The CMS pain parameter was not adjusted for BPI because they are similar measures.

We first checked that age, sex, pain severity, diagnosis, previous surgery, and work-related injury were not effect modifiers by subgroup analyses. After checking that the associations between predictors and outcomes did not differ between subgroups (non-significant interaction), we considered these variables as confounders.

Because the evolution in CMS score is also affected by the level of the score before treatment (i.e., patients with lower scores before treatment are more likely to show improvement than patients with already high baseline scores) [39], the evolution of the CMS during treatment was also adjusted for the baseline score. We did not adjust for social factors, because we did not find such associations with the CMS previously [23].

Standardized coefficients were computed for comparability of the scales. Because the HADS-D, PCS and TSK scores were positively correlated, we built the global psychological score (GPS) as their geometric mean to reduce the multi-collinearity risk in multivariable models. Building a geometric mean of the different scores has been used previously [23]. It basically consists of creating an interaction variable (multiplication of the variables). Nevertheless, coefficients of a regression model including a three-way interaction would be difficult to interpret, which is why we generated the GPS. Moreover, the aim of our study was not to compute the magnitude of the associations between the predictors (HADS-D, PCS, TSK) and our outcomes but simply to determine what parameters of the CMS were associated with the patient psychological factors. Given the number of predictors used, the 433 patients included were adequate. In subgroup analyses, the smallest group consisted of those with a diagnosis other than rotator cuff, with 126 patients, which allowed for including up to eight variables in regression models to retain a minimum of 15 observations per parameter [40]. The significance level was set at P < 0.05. All analyses were performed with Stata® 16.0 (Stata Corp, College Station, TX, USA).

## Results

### Demographics

**Fig. 1:**
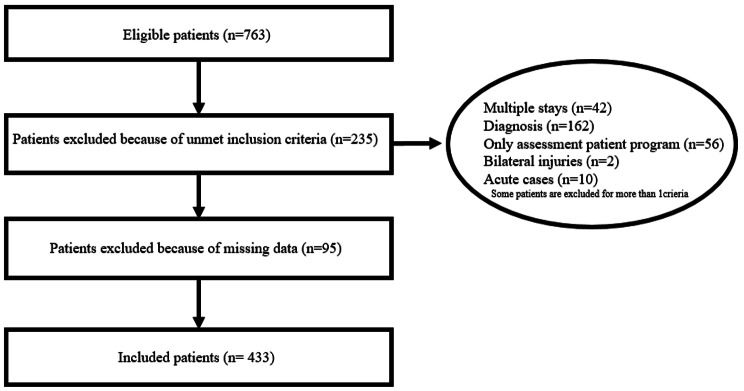
Flow chart of the selection of participants

We included 433 patients, mostly blue-collar workers (93%) and middle-aged males (88%, mean age 47 ± 11 years) with various shoulder injuries (75 [17.3%] adhesive capsulitis, 148 [34.2%] rotator cuff tear, 46 [10.6%], rotator cuff tendinopathy, 92 [21.3%] shoulder bursitis, 72 [16.6%] other diagnoses) and a median duration of symptoms of 392.2 days (interquartile range: 266.5-583.5). The flow chart shows the selection process (Fig. 1). Before treatment, patients had moderate pain (mean score 4.45 ± 1.99) and moderate scores for catastrophism and levels of kinesiophobia, and generally little to no depressive symptoms. The mean CMS at entry was 42.8 ± 15.5. The mean gain in score after treatment was 10.6 ± 10.9 (Table 1); for 48% of patients, the CMS improved by at least 10 points [15]. Patients excluded and included did not significantly differ in most measures (Table 1). The main differences between the two groups concerned the CMS scores at discharge (slightly worse evolution for excluded patients).

**Table 1:** Measures comparison of included versus excluded participants

Measure	Included (n = 433)		Excluded (n = 95)		N	P value
**Sex (categories)**	Men	Women	Men	Women	95 (100%)	
**Number of patients**	379 (88%)	54 (12%)	76 (80%)	19 (20%)		0.054
**Age (years)**	47 ± 11		48 ± 9		95 (100%)	0.699
**Rotator cuff issue (%)**	70.90		63.16		95 (100%)	0.138
**Fracture (%)**	15.70		21.05		95 (100%)	0.205
**Operated (%)**	68.82		65.26		95 (100%)	0.500
**Professional accident (%)**	60.05		63.16		95 (100%)	0.574
**BPI**	4.45 ± 1.99		4.75 ± 2.16		43 (45%)	0.370
**HADS-D**	6.73 ± 4.00		6.09 ± 4.07		45 (47%)	0.311
**PCS**	21.23 ± 12.07		20.74 ± 13.28		31 (33%)	0.828
**TSK**	44.38 ± 7.58		45.28 ± 9.10		46 (48%)	0.454
**entry**						
**CMS total**	42.76 ± 15.51		43.51 ± 18.27		88 (93%)	0.689
**CMS pain**	7.18 ± 2.90		7.12 ± 3.20			0.853
** CMS activity**	8.70 ± 3.44		9.14 ± 3.40			0.266
** CMS mobility**	21.68 ± 9.17		21.24 ± 10.40			0.685
** CMS strength**	5.22 ± 4.42		6.22 ± 5.62			0.068
**discharge**						
**CMS total**	53.31 ± 17.48		45.48 ± 19.50		65 (68%)	0.001
**CMS pain**	8.06 ± 3.19		7.39 ± 2.86			0.128
**CMS activity**	10.82 ± 3.77		9.31 ± 3.38			0.002
**CMS mobility**	26.79 ± 9.20		22.17 ± 10.38			< 0.001
**CMS strength**	7.74 ± 5.72		6.06 ± 6.42			0.030
**evolution**						
**CMS total**	10.55 ± 10.86		8.04 ± 9.27		64 (67%)	0.079
**CMS pain**	0.87 ± 2.65		1.09 ± 2.35			0.548
**CMS activity**	2.13 ± 2.69		1.25 ± 2.62			0.015
**CMS mobility**	5.11 ± 5.94		4.02 ± 7.28			0.185
**CMS strength**	2.52 ± 3.70		0.82 ± 4.19			< 0.001
**GPS**	17.38 ± 7.70		16.87 ± 8.83		30 (32%)	0.729
Quantitative values = mean ± SD.						

**CMS**, Constant Murley Score.  **BPI**, Brief Pain Inventory.  **HADS-D**, Hospital Anxiety and Depression Scale – Depression.  **PCS**, Pain Catastrophizing Scale.  **TSK**, Tampa Scale for Kinesiophobia.  **GPS**, Global Psychological Score = geometric mean HADS-D, PCS, TSK. **N**, number of excluded patients with available data

Before treatment, in the multivariable adjusted regression model, the GPS was significantly associated with only the pain CMS parameter: -0.37 (95% CI: -0.46 to -0.28), p <0.001 (Table 2). Details of the complete associations with confounding variables are in supplementary Tables 1 and 2.

**Table 2:** Linear regression models for Constant-Murley Score at **entry**

Outcome	Potential contributing factors	Univariable coefficient (95% CI)	P value	Potential contributing factors	Multivariable coefficient (95% CI)	P value
**Constant** **Sub-score pain**	**HADS-D**	-0.28 (-0.37 to -0.19)	< 0.001	**GPS**		
	**TSK**	-0.25 (-0.34 to -0.17)	< 0.001		-0.37 (-0.46 to -0.28)	< 0.001
	**PCS**	-0.40 (-0.48 to -0.31)	< 0.001			
**Constant** **Sub-score** **activity**	**HADS-D**	-0.08 (-0.17 to 0.02)	0.124	**GPS**		
	**TSK**	-0.08 (-0.18 to 0.01)	0.094		-0.07 (-0.17 to 0.03)	0.173
	**PCS**	-0.05 (-0.15 to 0.06)	0.374			
**Constant** **Sub-score** **mobility**	**HADS-D**	-0.11 (-0.21 to -0.02)	0.018	**GPS**		
	**TSK**	-0.04 (-0.13 to 0.06)	0.427		-0.07 (-0.17 to 0.03)	0.170
	**PCS**	-0.02 (-0.12 to 0.08)	0.677			
**Constant** **Sub-score** **strength**	**HADS-D**	-0.09 (-0.18 to -0.003)	0.043	**GPS**		
	**TSK**	-0.07 (-0.16 to 0.02)	0.132		-0.09 (-0.18 to 0.01)	0.078
	**PCS**	-0.07 (-0.16 to 0.03)	0.181			

**95% CI**, 95% confidence interval. **HADS-D**, Hospital Anxiety and Depression Scale – Depression.  **TSK**, Tampa Scale for Kinesiophobia.  **PCS**, Pain Catastrophizing Scale.  **GPS**, Global Psychological Score = geometric mean HADS-D, PCS, TSK. Models were adjusted for age, sex, diagnosis, surgery, work-related injury and pain severity (except for Constant sub-score pain)

After treatment, in the multivariable adjusted regression models, the GPS was associated with the evolution of the four CMS parameters: -0.12 (-0.23 to -0.01) to -0.26 (95% CI: -0.36 to -0.16), p < 0.05 (Table 3).

**Table 3:** Linear regression models for Constant -Murley Score **evolution**

Outcome	Potential contributing factors	Univariable coefficient (95% CI)	P value	Potential contributing factors	Multivariable coefficient (95% CI)	P value
**Constant** **Sub-score pain**	**HADS-D**	-0.18 (-0.28 to -0.08)	< 0.001	**GPS**		
	**TSK**	-0.17 (-0.26 to -0.07)	0.001		-0.26 (-0.36 to -0.16)	< 0.001
	**PCS**	-0.29 (-0.39 to -0.19)	< 0.001			
**Constant** **Sub-score** **activity**	**HADS-D**	-0.13 (-0.23 to -0.03)	0.010	**GPS**		
	**TSK**	-0.07 (-0.18 to 0.03)	0.146		-0.16 (-0.27 to -0.05)	0.003
	**PCS**	-0.14 (-0.25 to -0.03)	0.013			
**Constant** **Sub-score** **mobility**	**HADS-D**	-0.14 (-0.23 to -0.04)	0.005	**GPS**		
	**TSK**	-0.07 (-0.16 to 0.03)	0.169		-0.19 (-0.29 to -0.09)	< 0.001
	**PCS**	-0.16 (-0.27 to -0.06)	0.002			
**Constant** **Sub-score** **strength**	**HADS-D**	-0.09 (-0.19 to 0.01)	0.086	**GPS**		
	**TSK**	-0.05 (-0.15 to 0.05)	0.324		-0.12 (-0.23 to -0.01)	0.029
	**PCS**	-0.11 (-0.22 to 0.003)	0.057			

**95% CI**, 95% confidence interval. **HADS-D**, Hospital Anxiety and Depression Scale – Depression.  **TSK**, Tampa Scale for Kinesiophobia.  **PCS**, Pain Catastrophizing Scale.  **GPS**, Global Psychological Score = geometric mean HADS-D, PCS, TSK. Models were adjusted for age, sex, diagnosis, surgery, work-related injury and pain severity (except for Constant sub-score pain)

## Discussion

### Background and rationale

When they presented their clinical method of functional assessment of the shoulder, C.R. Constant and A.H. Murley said in 1987 [11] that “There is considerable controversy over an ideal method of functional assessment of the shoulder” [10, 41, 42]. More than 30 years have elapsed since this statement and the controversy is not yet over. Significant efforts have been made to improve the situation [36]. The notable development of PROMs has enabled patients to express their own feelings about their health and their treatment evolution, in contrast to clinician-rated tools.

Nevertheless, PROMs can be influenced by other factors such as perception during clinician assessment and the patient’s condition or treatment results, or especially psychosocial factors. They represent a weakness if we use them alone or as the main outcome measurement tool. A plethora of literature illustrates this [20, 43]. Constant and Murley proposed a method that included both patient- and clinician-rated outcomes [11]. However, this mix has been criticized from a methodological point of view, some finding it preferable to use different tools for different purposes [18] or at least to present the parameters of the CMS separately, and others questioning whether a tool like the CMS is free from the influence of psychosocial factors. One study found that the CMS was not correlated with psychologic measures [22] and others also found that functional improvement measured by the CMS was not related to anxiety or depressive symptoms after shoulder arthroscopy [44]. Recently, a systematic review obtained results against or inconclusive for the predictive value of psychological factors in patients with musculoskeletal disorders who underwent conservative intervention [45]. In contrast, in a previous study, we found that psychological factors (depression, catastrophism, and kinesiophobia) were related to functional improvement measured by the global CMS [23]. After adjustment for age, sex, pain severity, diagnosis, surgery, and work-related injury, the present results seem to confirm this association between the CMS and psychological factors. During a single cross-sectional measurement, only the pain parameter was related to these factors. When evaluating a clinical change during follow-up with at least two different measurements, all CMS parameters, not just the global score, were related to these factors.

### Parameters of the CMS associated with psychological factors before treatment

Our study shows that in a cross-sectional measurement of the CMS, only the “pain” parameter was negatively affected by psychological factors in the multivariable analysis. We cannot discuss this result in light of previous data because to our knowledge, this is the first study to investigate the parameters of CMS separately. Because the CMS is a composite score measured by adding up the four parameter scores, our results raise the question of whether the pain parameter should be measured separately from the other parameters of the CMS score. The pain parameter represents 15 points of the total score (100 points). Although the correlation between psychological factors and pain is not surprising [19, 44], we could have expected to find an association with the other parameter rated by the patient (i.e., activities of daily living). Why this association was not the case is difficult to determine based on this study alone. This parameter of the CMS is rather basic as compared with more recent functioning measures [13]. Moreover, this parameter derives half of its scoring from a question on functioning (work, recreation/sport, sleep) and another on the possible positioning to be reached with the hand. Therefore, this last item is “observable” by the clinician, which probably limits its purely subjective nature. Hence, all these points seem to represent an argument favoring those who consider that mixing patient- and clinician-rated outcomes is a weakness rather than a strength [18].

### Changes of parameters of the CMS associated with psychological factors

After treatment, despite the significant improvement in the total CMS (10.55 ± 10.86), the changes in the four CMS parameters were all negatively associated with psychological factors. For the same reason as above, comparisons cannot be made with previous studies. Nevertheless, this finding reinforces the results of our previous study, finding an association of the same psychological factors with the total CMS [23]. This observation emphasizes the need for all healthcare professionals who treat musculoskeletal disorders to consider psychological (emotional and cognitive) factors when using the CMS to evaluate the effect of a treatment [19, 21, 45–47]. A recent study showed that these factors did not prevent patients from perceiving improvement after shoulder surgery. However, functioning was lower at each assessment (baseline, 3 and 12 months), and the difference was above or close to the minimally clinically significant difference at all time points [48]. This finding should induce clinicians to actively seek these factors, not to excuse a disappointing result after treatment by invoking the patient’s psychological state, which would be tempting, but because there are effective techniques to deal with and improve the management [49, 50]. Some of these techniques are accessible to all healthcare professionals, including surgeons (i.e., to improve communication skills in discussing a diagnosis and in selecting treatment options and to promote the assessment and comprehensive management of the biopsychosocial determinants of health, all of which contribute to an effective clinician–patient relationship) [49]. Others require interdisciplinary management (cognitive-behavioural therapies, graded pain and activity exposure), and the best chance of success is to detect the patient could benefit early.

### Strengths

The strength of our study is highlighting the effect of psychological factors when assessing and monitoring chronic painful shoulder function with the CMS. Considering this relationship, clinicians should assess pain with another tool and be aware of this effect when following up patients, especially with clinical and imaging discrepancies. Our study also sensitizes clinicians to the search for depression, catastrophizing and kinesiophobia factors for a biopsychosocial management increasingly being used in chronic musculoskeletal pain [3, 51].

### Limitations

The first limitation is that our data are for patients with persistent shoulder pain treated in a rehabilitation teaching center specialized in the management of complex situations (failure of previous therapies, vocational aspects), so our results may not be generalizable to other patients or to other settings. Our population was about 10 years younger on average and the percentage of women (12%) lower (26–61%) as compared with the literature [43]. Nevertheless, we performed subgroup analyses. The confounders (age, sex, pain severity, diagnosis, previous surgery, and work-related injury) did not change the associations between the predictors and the outcomes.

An observational study also does not reveal whether a causal relationship exists. Hence, despite considerable efforts to include all eligible patients, we were unable to avoid some missing data. One of the reasons is the retrospective design of this study.

Excluded and included patients had similar profiles but a slightly less favorable evolution, without a simple explanation. Thus, the effect of other psychological factors could not be measured because they were not available in our database, especially patient expectation and pain self-efficacy, which seem to be important psychological factors related to the outcome [21, 48, 52, 53].

## Conclusions

This study suggests that the “pain parameter” was affected by the psychological factors studied (depression, catastrophism and kinesiophobia) when measuring the CMS, unlike the three other CMS parameters, which did not seem to be influenced by these factors. This finding raises the question of measuring “pain” separately when using the CMS score. The benefit would be to limit the effect of psychological factors and to have a measure that is as observable as possible. However, such a modification could require revalidating the CMS, which would be a major task. Because the evolution of all CMS parameters during follow-up was affected by the psychological factors investigated, the main conclusion of this study is that clinicians should be aware of this association when interpreting scores, which argues in favor of a biopsychosocial approach to patients with chronic shoulder pain, as is the case for low back pain [27]. In the future, the effect of other factors such as patient expectations and pain self-efficacy could be assessed.

## Electronic supplementary material

Below is the link to the electronic supplementary material.

Supplementary Material 1

## Data Availability

The datasets used and/or analysed during the current study are available from the corresponding author on reasonable request.
