# Supplementary material for: Should we separately measure the pain parameter of the Constant-Murley score in patients with chronic shoulder pain?
Source: BMC Musculoskelet Disord. 2023 May 19;24:399. doi: 10.1186/s12891-023-06441-7 (PMC10197235; doi:10.1186/s12891-023-06441-7)
Supplement: Supplementary file 1 — Supplementary Material 1 [file 12891_2023_6441_MOESM1_ESM.docx]

| Supplementary Table 1. Multivariable Linear regression models for Constant-Murley Score at **entry** | | | |
| --- | --- | --- | --- |
| **Outcome** | **Independent variable** | **Multivariable coefficient**  **(95% CI)** | **P value** |
| **Constant**  **Sub-score**  **pain** | **GPS** | -0.37  (-0.46 to -0.28) | <0.001 |
|  | **Age** | -0.00  (-0.01 to 0.00) | 0.371 |
|  | **Sex (female)** | -0.36  (-0.62 to -0.10) | 0.007 |
|  | **Surgery (yes)** | 0.12  (-0.07 to 0.30) | 0.214 |
|  | **Diagnosis (rotator cuff)** | -0.07  (-0.26 to 0.13) | 0.487 |
|  | **Work-related injury (yes)** | 0.07  (-0.10 to 0.25) | 0.415 |
| **Constant**  **Sub-score**  **activity** | **GPS** | -0.07  (-0.17 to 0.03) | 0.173 |
|  | **BPI (pain severity)** | -0.10  (-0.15 to -0.05) | <0.001 |
|  | **Age** | -0.01  (-0.01 to 0.00) | 0.259 |
|  | **Sex (female)** | -0.33  (-0.60 to -0.06) | 0.018 |
|  | **Surgery (yes)** | -0.12  (-0.30 to 0.09) | 0.281 |
|  | **Diagnosis (rotator cuff)** | 0.04  (-0.17 to 0.24) | 0.728 |
|  | **Work-related injury (yes)** | 0.01  (-0.18 to 0.19) | 0.941 |
| **Constant**  **Sub-score**  **mobility** | **GPS** | -0.7  (-0.17 to 0.03) | 0.170 |
|  | **BPI (pain severity)** | -0.07  (-0.12 to -0.02) | 0.008 |
|  | **Age** | -0.02  (-0.03 to -0.02) | <0.001 |
|  | **Sex (female)** | -0.11  (-0.38 to 0.16) | 0.437 |
|  | **Surgery (yes)** | -0.18  (-0.37 to 0.02) | 0.072 |
|  | **Diagnosis (rotator cuff)** | 0.09  (-0.12 to 0.29) | 0.406 |
|  | **Work-related injury (yes)** | -0.07  (-0.25 to 0.11) | 0.447 |
| **Constant**  **Sub-score**  **strength** | **GPS** | -0.09  (-0.18 to 0.01) | 0.078 |
|  | **BPI (pain severity)** | -0.04  (-0.09 to 0.00) | 0.064 |
|  | **Age** | -0.02  (-0.03 to -0.02) | <0.001 |
|  | **Sex (female)** | -0.55  (-0.80 to -0.29) | <0.001 |
|  | **Surgery (yes)** | -0.17  (-0.36 to 0.01) | 0.065 |
|  | **Diagnosis (rotator cuff)** | -0.11  (-0.30 to 0.08) | 0.271 |
|  | **Work-related injury (yes)** | -0.09  (-0.27 to 0.08) | 0.292 |
| **95% CI**, 95% confidence interval. **GPS**, Global Psychological Score = geometric mean HADS-D, PCS, TSK. **BPI**, Brief Pain Inventory. Models were adjusted for age, sex, diagnosis (rotator cuff vs. Others), surgery (Yes vs. No), work-related injury (Yes vs. No) and pain severity (except for Constant sub-score pain). | | | |

| Supplementary Table 2. Multivariable Linear regression models for Constant-Murley Score at **evolution** | | | |
| --- | --- | --- | --- |
| **Outcome** | **Independent variable** | **Multivariable coefficient**  **(95% CI)** | **P value** |
| **Constant**  **Sub-score**  **pain** | **GPS** | -0.26  (-0.36 to -0.16) | <0.001 |
|  | **Constant**  **Sub-score**  **pain at entry** | -0.46  (-0.56 to -0.36) | <0.001 |
|  | **Age** | 0.00  (-0.01 to 0.01) | 0.455 |
|  | **Sex (female)** | 0.01  (-0.26 to 0.29) | 0.921 |
|  | **Surgery (yes)** | -0.13  (-0.33 to 0.06) | 0.186 |
|  | **Diagnosis (rotator cuff)** | -0.10  (-0.31 to 0.10) | 0.330 |
|  | **Work-related injury (yes)** | -0.15  (-0.33 to 0.04) | 0.124 |
| **Constant**  **Sub-score**  **Activity** | **GPS** | -0.16  (-0.27 to -0.05) | 0.003 |
|  | **Constant**  **Sub-score**  **activity at entry** | -0.33  (-0.42 to -0.23) | <0.001 |
|  | **BPI (pain severity)** | -0.05  (-0.10 to 0.00) | 0.070 |
|  | **Age** | 0.00  (-0.00 to 0.01) | 0.301 |
|  | **Sex (female)** | 0.04  (-0.24 to 0.33) | 0.765 |
|  | **Surgery (yes)** | -0.36  (-0.56 to -0.16) | 0.001 |
|  | **Diagnosis (rotator cuff)** | -0.03  (-0.24 to 0.18) | 0.784 |
|  | **Work-related injury (yes)** | -0.19  (0.38 to -0.00) | 0.049 |
| **Constant**  **Sub-score**  **mobility** | **GPS** | -0.19  (-0.29 to -0.09) | <0.001 |
|  | **Constant**  **Sub-score**  **mobility at entry** | -0.38  (-0.48 to -0.28) | <0.001 |
|  | **BPI (pain severity)** | -0.03  (-0.08 to 0.02) | 0.180 |
|  | **Age** | -0.00  (-0.01 to 0.00) | 0.360 |
|  | **Sex (female)** | 0.03  (-0.24 to 0.30) | 0.816 |
|  | **Surgery (yes)** | -0.33  (-0.53 to -0.14) | 0.001 |
|  | **Diagnosis (rotator cuff)** | 0.14  (-0.07 to 0.34) | 0.186 |
|  | **Work-related injury (yes)** | 0.05  (-0.14 to 0.23) | 0.625 |
| **Constant**  **Sub-score**  **strength** | **GPS** | -0.12  (-0.23 to -0.01) | 0.029 |
|  | **Constant**  **Sub-score**  **strength at entry** | -0.11  (-0.22 to -0.01) | 0.034 |
|  | **BPI (pain severity)** | 0.01  (-0.05 to 0.06) | 0.837 |
|  | **Age** | -0.01  (-0.02 to -0.00) | 0.005 |
|  | **Sex (female)** | -0.45  (-0.74 to -0.16) | 0.003 |
|  | **Surgery (yes)** | -0.14  (-0.35 to 0.06) | 0.167 |
|  | **Diagnosis (rotator cuff)** | -0.18  (-0.40 to 0.03) | 0.096 |
|  | **Work-related injury (yes)** | -0.03  (-0.22 to 0.17) | 0.778 |
| **95% CI**, 95% confidence interval. **GPS**, Global Psychological Score = geometric mean HADS-D, PCS, TSK. **BPI**, Brief Pain Inventory. Models were adjusted for age, sex, diagnosis (rotator cuff vs. Others), surgery (Yes vs. No), work-related injury (Yes vs. No) and pain severity (except for Constant sub-score pain). | | | |
